# Nobiletin Alleviates Astrocyte Activation and Oxidative Stress Induced by Hypoxia In Vitro

**DOI:** 10.3390/molecules27061962

**Published:** 2022-03-17

**Authors:** Dandan Wang, Fengjuan Gao, Fangyuan Hu, Jihong Wu

**Affiliations:** 1 Eye Institute, Eye and ENT Hospital, College of Medicine, Fudan University, Shanghai 200030, China; aiyawy@126.com (D.W.); gaofengjuan0815@sina.com (F.G.); huyaya_003@163.com (F.H.); 2 Shanghai Key Laboratory of Visual Impairment and Restoration, Science and Technology Commission of Shanghai Municipality, Shanghai 200030, China; 3 Key Laboratory of Myopia, Ministry of Health, Shanghai 200030, China

**Keywords:** nobiletin, astrocyte, neuroprotective effect, oxidative stress, mitochondria

## Abstract

Increasing evidence indicates that nobiletin (NOB) is a promising neuroprotective agent. Astrocyte activation plays a key role in neurodegenerative disorders. Thus, this study aims to investigate the effects of NOB on astrocyte activation and the potential mechanisms. In this study, astrocytes were exposed to hypoxia injury for 24 h to induce activation in vitro. Glial fibrillary acidic protein (GFAP) was chosen as a marker of astrocyte activation. To evaluate the effects of NOB on the migration of activated astrocytes, we used a scratch wound healing assay and Transwell migration assay. In addition, the levels of reactive oxygen species (ROS), malondialdehyde (MDA), mitochondrial membrane potential, Nrf2 and HO-1 were measured to investigate the mechanisms of NOB in the activation of astrocytes. We found that NOB alleviated astrocyte activation and decreased GFAP expression during hypoxia. Simultaneously, NOB alleviated the migration of astrocytes induced by hypoxia. With NOB treatment, hypoxia-induced oxidative stress was partially reversed, including reducing the production of ROS and MDA. Furthermore, NOB significantly improved the mitochondrial dysfunction in activated astrocytes. Finally, NOB promoted Nrf2 nuclear translocation and HO-1 expression in response to continuous oxidative damage. Our study indicates, for the first time, that NOB alleviates the activation of astrocytes induced by hypoxia in vitro, in part by ameliorating oxidative stress and mitochondrial dysfunction. This provides new insights into the neuroprotective effects of NOB.

## 1. Introduction

Nerve injury is one of the most common clinical manifestations, usually observed in ischemia and trauma. It is characterized by the death of neuron and axon degeneration. Currently, there is no effective treatment to reverse the damage. After nerve injury, reactive astrogliosis occurs, including the activation and migration of astrocytes, which is associated with axon damage [1]. In addition, the glial scar, composed mainly of astrocytes, is widely recognized as the major obstacle to axon regeneration [2,3]. Recently, it has been reported that reactive astrocytes play a key role in extracellular matrix (ECM) remodeling of the nerve by influencing the synthesis and deposition of ECM [4]. When neuropathy occurs, activated astrocytes can reshape ECM, creating an environment that is not conducive to axon regeneration [5]. Therefore, alleviating astrocyte activation is a potential neuroprotective strategy that can be used to reduce axon damage and promote axon regeneration.

Nobiletin (NOB) is a polymethoxylated flavone derived from the peels of citrus fruits [6]. In recent years, NOB has attracted increasing attention for its variety of potential health benefits. Several studies in vivo and in vitro have demonstrated that NOB exerts favorable effects that are anti-inflammatory [7], anti-oxidation [8], anti-aging [9], and the improvement on endoplasmic reticulum (ER) stress [10] and mitochondrial dysfunction [11]. Increasing evidence suggests that NOB is a promising molecule that can be used to prevent neurodegenerative and neurological diseases. In animal models of Alzheimer’s disease (AD), NOB has been shown to, respectively, improve short-term memory and cognitive impairment by reducing the soluble Aβ levels and scavenging the reactive oxygen species (ROS) [12]. Besides, the treatment of nobiletin was found to ameliorate motor impairment and cognitive deficits, and protect dopaminergic neurons from neurotoxicity in animal models of Parkinson’s disease (PD) [13,14]. Furthermore, NOB also displays an outstanding antioxidative ability in ischemic injury models. NOB treatment reduced brain edema and neurological deficits, and decreased the infarct volume at 24 h after ischemic stroke [15]. Nevertheless, the effects of NOB on activated astrocytes during neuropathy remain unclear.

As is well known, oxidative stress takes an important part in the process of neuropathy [16]. To investigate the effects of NOB on activated astrocyte and further explore the related mechanisms, we used a hypoxia model to mimic the conditions for neuropathy to activate astrocytes in vitro. The results of this study provide new insights into the neuroprotective effects of NOB, involving the inhibition of astrocyte activation.

## 2. Materials and Methods

### 2.1. Drugs and Reagents

Nobiletin (LCMS purified, 99.04%; dissolved in dimethyl sulfoxide, final dose 0.0005% *v*/*v*) was obtained from MedChemExpress (Jersey City, NJ, USA). The reagents and materials for each kit are listed with each method.

### 2.2. Cell Culture and Hypoxia Model

Rat astrocytes were purchased from American Type Culture Collection (ATCC) and were cultured in DMEM/F12 supplemented with 10% fetal bovine serum (FBS) and 1% penicillin-streptomycin at 37 °C and 5% CO_2_. The medium was replaced every 48 h. Briefly, astrocytes were cultured under normal conditions for 24 h, and then placed under hypoxic conditions (0.2% O_2_, 94.8% N_2_, 5% CO_2_) at 37 °C for 12 h, 24 h and 48 h to induce hypoxic injury. Moreover, astrocytes were incubated with 1 μM NOB under hypoxia as NOB treatment group.

### 2.3. Protein Expression Analysis

Astrocytes were lysed in ice-cold RIPA buffer (Beyotime, Shanghai, China) and protease inhibitor cocktail (Roche, Mannheim, Germany). The protein concentration was quantified using a BCA protein assay kit (Beyotime, Shanghai, China). The Simple Western system (WES) (ProteinSimple, San Jose, CA, USA) was used to quantify the level of the glial fibrillary acidic protein (GFAP). Briefly, the sample was mixed with 0.1× Sample buffer and fluorescent 5× Master Mix (ProteinSimple), and then denatured at 95 °C for 5 min. The prepared samples, biotinylated ladder, blocking solution, primary antibodies, secondary antibodies, secondary streptavidin-HRP, chemiluminescent reagent and wash buffer (ProteinSimple) were loaded on the microplates according to the manufacturer’s protocol. Then, the subsequent procedures, including protein separation, washing, immunodetection and data analysis, were performed automatically by the machine. The chemiluminescent images were produced and analyzed by the Compass software (version 6.0, ProteinSimple). Primary antibodies against GFAP (1:50 dilution, Cell Signaling Technology, MA, USA), β-actin (1:100 dilution, Cell Signaling Technology), Nrf2 (1:50 dilution, Abcam, Cambridge, UK), HO-1 (1:50 dilution, Cell Signaling Technology) and Histone H3 (1:250 dilution, Cell Signaling Technology) were used in the experiment. The results are shown as the fold intensity relative to the control group.

### 2.4. Scratch Wound Healing Assay

Astrocytes were seeded in 6-well plates at a density of 2 × 10^5^ cells per well. When the cell monolayer was formed, a 200 μL tip was used to introduce a linear wound gap in the center of the layer. Then, the suspended cells were washed away with PBS. The attached cells were treated with hypoxia, or both hypoxia and NOB (1 μm), and starved in a culture medium without FBS for 24 h. The wound healing at 0, 12 and 24 h was photographed using a Leica DMI 3000B microscope (×10), and the wound area at different time points was measured using ImageJ. The assay was performed in triplicate per separate experiment. The results are expressed as the ratios of the migration area to the beginning area.

### 2.5. Transwell Migration Assay

After hypoxia with or without NOB (1 μm), astrocytes were subsequently detached by 0.25% trypsin. Transwell chambers with an 8 µm pore size (Corning, NY, USA) were used. Approximately 1 × 10^4^ astrocytes in serum-free medium were added into the upper chambers and the lower chambers were filled with culture medium containing 10% FBS. After migration for up to 24 h, astrocytes underneath the membrane were fixed with ice-cold absolute methyl alcohol for 15 min, and then stained with 0.5% crystal violet for another 15 min. The unmigrated astrocytes were wiped away. Five random fields of each chamber were selected to count the number of the migrated astrocytes with a Leica DMI 3000B microscope (×10), and the mean values were calculated. The assay was performed in triplicate per separate experiment.

### 2.6. ROS Detection

Approximately 8 × 10^3^ astrocytes were seeded in 96-well plates. The levels of ROS in astrocytes were detected using an ROS Assay Kit (Beyotime, Shanghai, China). Briefly, the pre-treated astrocytes were incubated with 2′,7′-Dichlorodihydrofluorescein diacetate (DCFH-DA) for 20 min at 37 °C, and then gently washed three times with PBS. The images were captured using a Leica DMI 3000B microscope (×10), and the fluorescence intensities were measured using ImageJ. The assay was performed in triplicate per separate experiment.

### 2.7. Malondialdehyde (MDA) Detection

The pre-treated astrocytes were collected and homogenized in ice-cold RIPA buffer (Beyotime, Shanghai, China), and then centrifuged at 10,000× *g* for 10 min at 4 °C to obtain the supernatants. To assess the levels of MDA in astrocytes, the MDA Assay Kit (Beyotime, Shanghai, China) was performed according to the manufacturer’s protocol, and then the reactive substances were measured at 532 nm with a microplate reader (Tecan; Infnite M1000, Männedorf, Switzerland). The results were normalized with protein content using a BCA protein assay kit (Beyotime, Shanghai, China), and are presented as fold activities to the control group.

### 2.8. Mitochondrial Membrane Potential Detection

Astrocytes were seeded in 96-well plates at a density of 8 × 10^3^ cells per well. Mitochondrial membrane potential was detected using a JC-1 Assay Kit (Beyotime, Shanghai, China). In brief, the pre-treated astrocytes were incubated with JC-1 for 20 min at 37 °C, and then gently rinsed twice with wash buffer. The fluorescence intensities of red aggregates and green monomers were measured by a microplate reader (Tecan; Infnite M1000, Männedorf, Switzerland), and the ratio was calculated to elucidate the mitochondrial membrane potential. The images were obtained by a Leica DMI 3000B microscope (×20). The assay was performed in triplicate per separate experiment.

### 2.9. Immunofluorescence

Approximately 1 × 10^4^ astrocytes were seeded in 24-well plates. After 24 h of hypoxia, the pre-treated astrocytes were fixed with paraformaldehyde for 30 min, and then cells were permeabilized with 0.3% Triton X-100 for 15 min and blocked with 3% bovine serum albumin (BSA) for 1 h. Subsequently, cells were incubated with primary antibody against rabbit anti-Nrf2 (1:200, Abcam) at 4 °C, overnight. After washing with PBS for three times, cells were incubated with Alexa Fluor 555-conjugated donkey anti-rabbit secondary antibody (1:800, Invitrogen, CA, USA) at room temperature for 1 h. The cell nuclei were stained with DAPI. The images were obtained using a Leica microscope (×20). The assay was done in triplicate per separate experiment.

### 2.10. Cell Viability

Approximately 8 × 10^3^ astrocytes were seeded in 96-well plates under normal conditions for 24 h. Then, astrocytes were treated with or without NOB (1 μm) under hypoxia for another 24 h. The cell viability was examined by the Cell Counting Kit-8 (CCK-8) (Dojindo Laboratories, Kumamoto, Japan) according to the manufacturer’s protocol. The optical density values were measured at 450 nm using a microplate reader (Tecan; Infnite M1000, Männedorf, Switzerland), and the results are shown as ratios to the control values.

### 2.11. Statistical Analysis

Each experiment was repeated at least three times. The data are presented as means ± standard deviation, and were analyzed statistically with a One-way analysis of variance with a Bonferroni correction using GraphPad Prism 8.0 (San Diego, CA, USA). Lastly, a *p* value < 0.05 was defined as statistically significant.

## 3. Results

### 3.1. NOB Alleviates the Activation of Hypoxia-Induced Astrocytes

**Figure 1:**
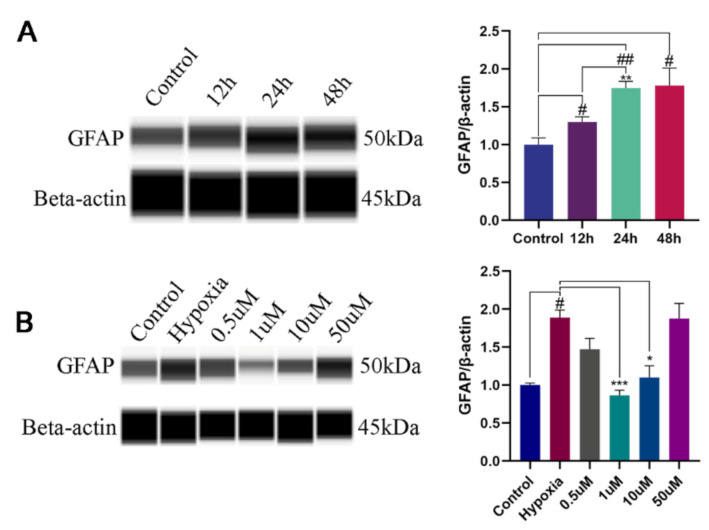
The levels of GFAP expression in astrocytes by WES. (**A**) The levels of GFAP expression in astrocytes after hypoxia of 12 h, 24 h and 48 h, and the corresponding statistical analysis. # *p* < 0.05 and ## *p* < 0.01 vs. control, ** *p* < 0.01 vs. 12 h hypoxia group. (**B**) Effects of different NOB concentrations on GFAP expression in astrocytes after hypoxia of 24 h, and the corresponding statistical analysis. The data are shown as mean ± SD, *n* = 3, # *p* < 0.05 vs. control, * *p* < 0.05 and *** *p* ≤ 0.001 vs. 24 h hypoxia group.

The upregulation of GFAP is considered as a marker of astrocyte reactivity [17]. To assess whether NOB could alleviate the activation of hypoxia-induced astrocytes, the level of GFAP expression was measured. After 12 h, 24 h and 48 h of hypoxia, the protein expression significantly increased in a time-dependent manner compared with the control group (Figure 1A; *p* = 0.0379, 0.0021 and 0.0438, respectively; *n* = 3), but there was no significant difference between the 24 h and 48 h group (*p* = 0.9998; *n* = 3). Therefore, 24 h of hypoxia was used to induce the activation of astrocytes in this study.

To investigate the effects of NOB on activated astrocyte, cells were treated with different concentration of NOB during hypoxia. As shown in Figure 1B, the upregulation of GFAP was markedly attenuated by the administration of 1 μm and 10 μm NOB during the course of hypoxia (*p* = 0.0010 and 0.0309, respectively; *n* = 3). However, no statistical difference was found between the two groups (*p* = 0.4569; *n* = 3). The results indicate that NOB treatment can alleviate the astrocyte activation induced by hypoxia. According to the above results, we used 1 μM NOB to conduct the subsequent experiments.

### 3.2. NOB Inhibits the Migration of Hypoxia-Induced Astrocytes

**Figure 2:**
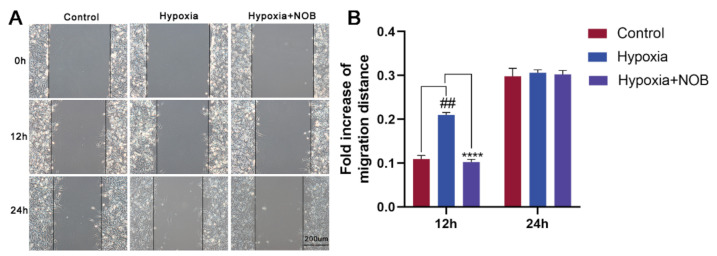
NOB inhibits hypoxia-induced astrocyte migration in scratch wound healing assay. (**A**) The images of wound healing at 0, 12 and 24 h in different groups. Scale bar, 200 μm. (**B**) Statistical analysis of migration area. The data are expressed as mean ± SD, *n* = 3. ## *p* < 0.01 vs. control, **** *p* < 0.0001 vs. hypoxia group.

Previous research has shown that reactive astrogliosis, including astrocyte activation and migration, occurs upon injury [18]. To explore the influence of NOB on astrocyte migration induced by hypoxia, scratch wound healing assay was performed in this study. As shown in Figure 2, astrocyte migration increased significantly after 12 h of hypoxia compared with the control (*p* = 0.0017, *n* = 3), which was reversed in the astrocytes supplemented with NOB during hypoxic conditions (*p* < 0.0001, *n* = 3). However, there was no significant difference in the area of migration after 24 h between the control group and hypoxia group (*p* = 0.9501, *n* = 3). This may be related to the severe hypoxia, and being serum-free for 24 h, which caused excessive damage to astrocytes.

**Figure 3:**
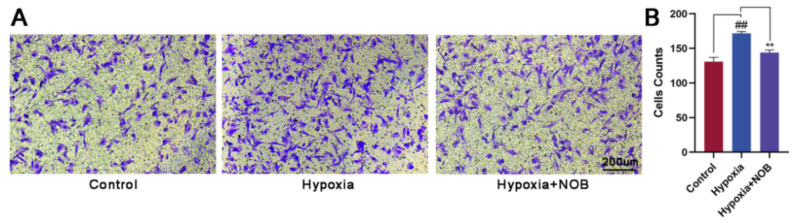
NOB inhibits hypoxia-induced astrocyte migration in Transwell migration assay. (**A**) The images of migrated astrocytes in different groups after 24 h. Scale bar, 200 μm. (**B**) Statistical analysis of the number of migrated astrocytes. The values are expressed as mean ± SD, *n* = 3, ## *p* < 0.01 vs. control, ** *p* < 0.01 vs. hypoxia group.

To further verify the effect of NOB on the cellular migration, we also performed a Transwell migration assay. Compared with the control group, a marked increase in the number of migrated cells was observed under hypoxia (Figure 3; *p* = 0.0046, *n* = 3). With the presence of NOB during the course of hypoxia, the change was largely attenuated (*p* = 0.0012, *n* = 3). Considering these results together, we can reach a conclusion that astrocyte activation is strongly alleviated by NOB treatment under hypoxia, which is indicated by the reduced GFAP expression and the slow cellular migration.

### 3.3. NOB Reduces Oxidative Stress in Activated Astrocytes

NOB, as a kind of polymethoxylated flavone, has shown to be potentially neuroprotective in neurodegenerative diseases, such as AD and PD, which is related to its antioxidant capacity [19]. Therefore, we speculated that the inhibitory effect of NOB on activated astrocytes might also be associated with oxidative stress. To test our hypothesis, we measured two relevant biomarkers, including ROS and MDA.

**Figure 4:**
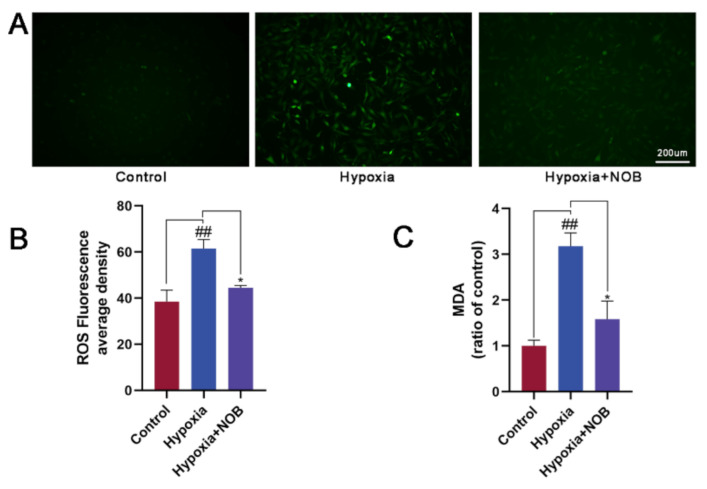
NOB treatment shows strong antioxidant effects on astrocytes activated by hypoxia for 24 h, and reduces the intracellular ROS and MDA levels. (**A**) The ROS fluorescent images of astrocytes in different groups. Scale bar, 200 μm. (**B**) Quantitative analysis of ROS fluorescence intensities in different groups. (**C**) Statistical analysis of intracellular MDA levels in different groups. The values are shown as mean ± SD, *n* = 3, ## *p* < 0.01 vs. control, * *p* < 0.05 vs. hypoxia group.

As shown in Figure 4A,B, a significant increase in the mean ROS fluorescence intensity was observed in activated astrocytes by their constant exposure to hypoxic environment, relative to the control (*p* = 0.0084, *n* = 3). Nevertheless, it was largely reversed by the administration of NOB in the course of hypoxia (*p* = 0.0380, *n* = 3), which confirmed the capacity of NOB in improving hypoxia-induced oxidative stress. In addition, we found that the level of MDA was markedly higher in activated astrocytes after 24 h of hypoxia than the control level (Figure 4C; *p* = 0.0030, *n* = 3). Nevertheless, the change was attenuated by the simultaneous exposure of the astrocytes to NOB during hypoxia (*p* = 0.0127, *n* = 3). These results suggest that the inhibitory effect of NOB on astrocyte reactivity may be mediated, at least in part, by the antioxidant mechanism of ROS and MDA clearance.

### 3.4. NOB Ameliorates Mitochondrial Dysfunction of Activated Astrocytes

**Figure 5:**
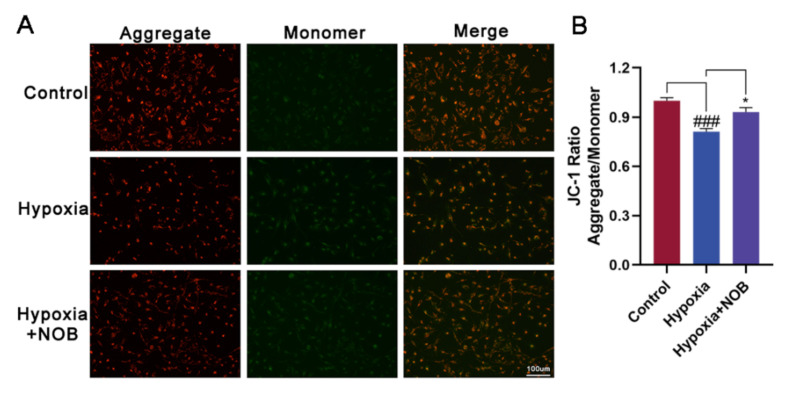
NOB ameliorates mitochondrial dysfunction in activated astrocytes induced by hypoxia for 24 h. (**A**) The JC-1 fluorescent images of astrocytes in different groups. Scale bar, 100 μm. (**B**) Quantitative analysis of JC-1 fluorescence intensities in astrocytes in different groups. The data are presented as mean ± SD, *n* = 3, ### *p* < 0.001 vs. control, * *p* < 0.05 vs. hypoxia group.

Several studies have demonstrated that NOB plays an important role in modulating mitochondrial function, including influencing the mitochondrial membrane potential [20,21,22,23]. To explore whether NOB can improve the mitochondrial function in activated astrocytes induced by hypoxia, we detected the mitochondrial membrane potential using a JC-1 Assay Kit. As shown in Figure 5B, hypoxia caused mitochondrial damage in activated astrocytes, resulting in a reduced mitochondrial membrane potential. Relative to the control, the difference was statistically significant (*p* = 0.0006, *n* = 3). Following the treatment of NOB during hypoxia, the change was largely reversed (*p* = 0.0206, *n* = 3). The fluorescent images show that the fluorescence intensities of red aggregates decreased while those of green monomers increased in activated astrocytes. Of note, NOB treatment attenuated the changes (Figure 5A). These results indicate that NOB helps to ameliorate the mitochondrial dysfunction of activated astrocytes.

### 3.5. NOB Promotes Nrf2 Nuclear Translocation and HO-1 Upregulation

**Figure 6:**
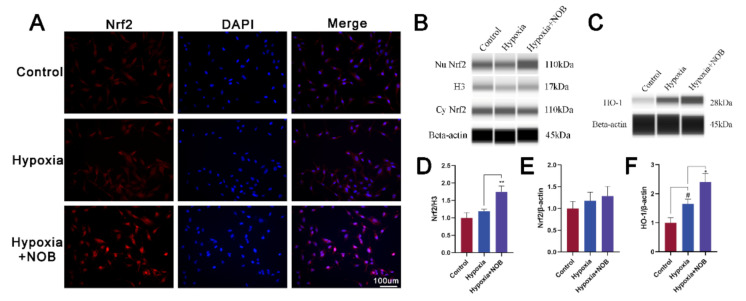
NOB promotes Nrf2 nuclear translocation and HO-1 upregulation in activated astrocytes induced by hypoxia for 24 h. (**A**) The Nrf2 immunofluorescence images of astrocytes in different groups. Scale bar, 100 μm. (**B**) The levels of nuclear and cytoplasmic Nrf2 expression in astrocytes after hypoxia of 24 h. (**C**) The levels of HO-1 expression in astrocytes after hypoxia of 24 h. (**D**) Quantification of nuclear Nrf2 normalized to H3. (**E**) Quantification of cytoplasmic Nrf2 normalized to β-actin. (**F**) Quantification of HO-1 normalized to β-actin. The data are presented as mean ± SD, *n* = 3, # *p* < 0.05 vs. control, * *p* < 0.05 and ** *p* < 0.01 vs. hypoxia group.

As is known, nuclear factor erythroid 2-related factor 2 (Nrf2) is an important antioxidant regulator. In conditions of oxidative stress, Nrf2 is transferred into the nucleus and subsequently activates the expression of antioxidant enzymes, such as heme oxygenase 1 (HO-1) [24]. To explore the exact molecular mechanism of NOB reducing oxidative stress, we detected the level of Nrf2 and HO-1, as well as Nrf2 immunofluorescence. As shown in Figure 6A, the accumulation of Nrf2 immunofluorescence in the nuclei of astrocytes increased with NOB treatment. Furthermore, it is also supported by quantification of the nuclear Nrf2 protein (Figure 6B; *p* = 0.0056, *n* = 3). Compared with the control, the level of HO-1 increased after 24 h of hypoxia (Figure 6C; *p* = 0.0263, *n* = 3). Of note, NOB administration further upregulated the expression of HO-1 (*p* = 0.0135, *n* = 3). Above all, NOB promotes the nuclear translocation of Nrf2 and subsequently upregulates the expression of HO-1 in activated astrocytes.

### 3.6. The Effect of NOB on Cell Viability

**Figure 7:**
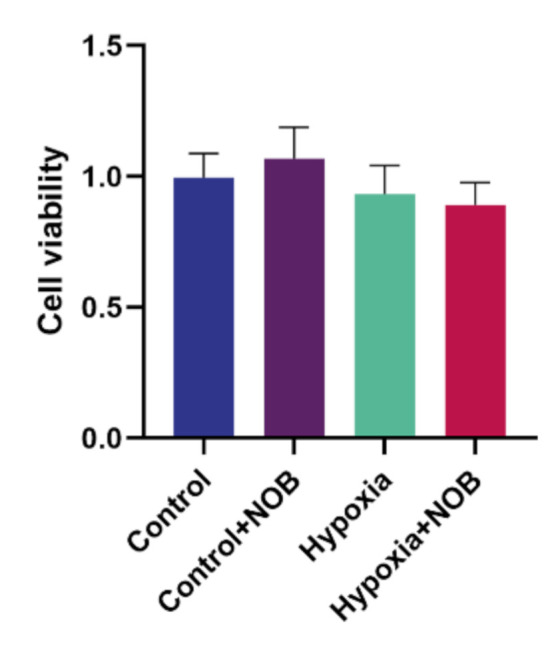
Cell viability of astrocytes treated with or without 1 μm NOB under normal conditions and hypoxic conditions by the CCK-8 assay. The data are shown as mean ± SD, *n* = 3.

Compared with the control level, the average viability of astrocytes was slightly decreased after 24 h of hypoxia. However, the difference was not statistically significant (Figure 7; *p* = 0.8730, *n* = 3). In addition, no apparent difference in cell viability was observed under hypoxic conditions with or without NOB treatment (*p* = 0.9591, *n* = 3). Although cell viability slightly improved under normal conditions after the treatment of NOB, there was no significant difference relative to the control group (*p* = 0.8238, *n* = 3). These results suggested that NOB alleviates hypoxia-induced astrocyte activation without influencing the cell viability and 1 μm NOB is nontoxic for astrocytes. Moreover, NOB does not affect the cell viability of astrocytes under normal conditions.

## 4. Discussion

Astrocytes are the major glial cells in the central nervous system (CNS), and play a critical role in the pathology of CNS. Due to their high sensitivity to changes in the microenvironment, astrocytes undergo a strong phenotypic transformation when damage occurs [18]. This process is called reactive astrogliosis, involving the activation and migration of astrocytes. In addition, previous research has indicated that it plays a key role in the secondary injury of CNS pathologies, including oxidative stress [25] and inflammation [26]. Therefore, the inhibition of astrocyte reactivity may be a potential neuroprotective target. Oxidative stress and hypoxia have been proven to play a critical role in the process of neurodegeneration in various neurological disorders, such as amyotrophic lateral sclerosis (ALS), PD and AD [27,28,29]. Oxidized free radicals at high concentrations are extremely reactive and damage neurons in CNS [30]. It also results in the dysfunction of activated astrocytes [25]. NOB is a polymethoxylated flavone and displays an excellent ability in antioxidant and anti- inflammatory in vivo and in vitro [31,32]. It is regarded as a promising therapy for alleviating neuronal degeneration. In this study, we indicate for the first time that NOB can alleviate astrocyte activation induced by hypoxia by reducing oxidative stress and improving mitochondrial dysfunction.

Consistent with previous research [33,34], hypoxia could induce the activation of astrocytes with the upregulation of GFAP expression. Notably, we found that GFAP expression significantly decreased in activated astrocytes with the administration of NOB during hypoxia. This suggests that NOB treatment alleviates astrocyte activation under hypoxic conditions. In line with a previous study [35], the results of this study indicated that NOB treatment did not affect cellular viability in either an anoxic or normal environment. Therefore, it is suggested that the inhibition of NOB on hypoxia-induced astrocyte activation is not the outcome of cytotoxic effects.

In response to damage, activated astrocytes would migrate to the site of injury and form a glial scar to surround the lesions [36]. In this study, we observed a faster migration rate of astrocytes after 24 h of hypoxia treatment than the control group using Transwell migration assay. This result is consistent with previous studies [37]. Nevertheless, the administration of NOB during hypoxia reversed the effect. Similarly, the migration of activated astrocytes was enhanced after 12 h of hypoxia treatment relative to the control in the scratch wound healing assay, and the change was attenuated by NOB treatment. Astrocyte migration is a critical step in the formation of glial scar and tissue remodeling [2]. Based on these findings, we infer that NOB treatment may help to reduce reactive astrocyte aggregation and glial scar formation at the injury site. However, the mechanisms that regulate the migration of activated astrocytes remain unclear [5]. Hence, further exploration is necessary. A better understanding of the mechanisms can lead to the development of new therapeutic strategies to reduce lesion size and promote repair in neurological diseases.

As is known, ROS has been involved as a key factor in neurodegenerative disorders, such as AD and PD [38,39]. When ROS production exceeds the cellular clear capacity, oxidative stress occurs, resulting in oxidative injury and cell death. MDA is another common biomarker of oxidative stress. It is the result of reactions between ROS and lipids, and is therefore affected by the overproduction of ROS [40]. In this study, the levels of ROS and MDA significantly increased in activated astrocytes induced by hypoxia. After NOB treatment during the course of hypoxia, the levels of ROS and MDA were remarkably decreased. It is suggested that NOB treatment can attenuate oxidative stress in activated astrocytes under hypoxic conditions. Previous research has shown that the mitochondrial respiratory chain is the main source of intracellular ROS [41]. As an organ with high energy requirements, CNS is extremely dependent on the mitochondria and their components [42]. When mitochondrial dysfunction occurs, oxidative stress is further exacerbated, followed by neuronal dysfunction and even death [43]. Mitochondrial membrane potential is a valuable indicator, reflecting the functional status of mitochondria in living cells. In this study, a decreased mitochondrial membrane potential was observed in hypoxia-induced activated astrocytes, while NOB treatment attenuated this change. Taken together, it demonstrated that NOB is a drug that targets mitochondrion, which can significantly enhance mitochondrial function and exhibits strong protective effects on astrocytes during hypoxia. Consistent with our study, Amarsanaa et al. [20] found NOB could regulate mitochondrial membrane potential through the K^+^ channel, and improve mitochondrial dysfunction by activating antioxidant signaling cascades. Thus, we infer that NOB alleviates astrocyte activation induced by hypoxia by ameliorating oxidative stress and mitochondrial dysfunction, to facilitate a stable transition between resting and active states of astrocytes in stress conditions.

Additionally, Nrf2 is thought to be the “master regulator” of antioxidant responses, regulating the expression of downstream antioxidant genes [44]. Recently, increasing evidence has demonstrated that Nrf2 is a potential therapeutic target for neurodegenerative disorders [44,45]. Under homeostatic conditions, Nrf2 is sequestered in the cytoplasm by binding to Kelch-like ECH associated protein 1 (Keap1). When oxidative stress occurs, Keap1 undergoes conformational changes followed by the release of Nrf2, which is then transferred into the nucleus and subsequently activates the expression of antioxidant enzymes, such as HO-1 [24]. Here, we found that NOB treatment promoted the nuclear translocation of Nrf2 in activated astrocytes, thereby reducing oxidative damage. Furthermore, HO-1 is a crucial antioxidant enzyme downstream of Nrf2. Previous research has shown that exposure to hypoxia can induce the expression of HO-1 [46]. Similarly, we observed an increased level of HO-1 in astrocytes by the constant exposure to a hypoxic environment. It may be an endogenous antioxidant response. However, this compensatory mechanism may begin to fail with the cumulative damage of hypoxia. With the treatment of NOB, the expression of HO-1 was further enhanced to resist oxidative damage.

In conclusion, our study indicates that NOB alleviates astrocyte activation induced by hypoxia in vitro by improving oxidative stress and enhancing mitochondrial function, as well as by increasing Nrf2 nuclear translocation and HO-1 upregulation. This presents a novel insight into the neuroprotective effects of NOB. NOB is a promising neuroprotective drug, so it is necessary to further investigate its exact neuroprotective mechanism and clarify its role in neurodegenerative disorders.

## Data Availability

Not applicable.

## Sample Availability

Samples of the compounds studied are unavailable from the authors for distribution.
